# Report on the Practical Operation of the Law Relating to the Importation of Adulterated and Spurious Drugs, &c.

**Published:** 1849-11

**Authors:** 


### ARTICLE X.

*Report on the Practical Operation of the Law relating to the Importation of Adulterated and Spurious Drugs, Medicines, &c.*—By M. J. Bailey, M.D.; Special Examiner of that class of Merchandise in the United States Customs at the Port of New-York. Read before the New-York Academy of Medicine, June 6th, 1849. Published by order of the Academy. From the New-York Journal of Medicine.—(From the Author.)

The law relating to the importation of adulterated drugs has been productive of very important results, as will be seen from the following catalogue of rejected articles, on page 5 of this report:

*Regne Animal, tom. i. p. 63.

| | | | |
|---|---|---|---|
| July, 1848, | 7,581 lbs. | Rhubarb root, | from Canton. |
| August, | 750 lbs. | Opium, | do. Marsailles. |
| do. | 2,940 lbs. | Jalap root, | do. Tampico, |
| do. | 2,249 lbs. | Rhubarb root, | do. London. |
| September, | 646 lbs. | do. do. | do. do. |
| do. | 1,414 lbs. | Gamboge, | do. do. |
| do. | 646 lbs. | Rhubarb, | do. Hamburg. |
| do. | 1,400 lbs. | Senna, | do. Leghorn. |
| do. | 2,900 lbs. | Spurious Yellow Bark, | do. Bordeaux. |
| do. | 875 lbs. | Rhubarb, | do. Canton, |
| do. | 758 lbs. | Opium, | do: London. |
| do. | 1,783 oz. | Iodine, | do. do. |
| do. | 1,075 lbs. | Rhubarb, | do. Marsailles. |
| do. | 4,275 lbs. | Jalap, | do. Vera Cruz. |
| October, | 788 lbs. | Rhubarb, | do. London. |
| do. | 227 lbs. | Myrrh, | do. do. |
| do. | 13,120 lbs. | Spurious Yellow Bark, | do. Maracaibo. |
| do. | 1,875 lbs. | do. do. do. | do. Bordeaux, |
| November, | 412 lbs. | Myrrh, | do. London, |
| do. | 1,280 oz. | Iodine, | do. Glasgow, |
| do. | 860 lbs. | Opium, | do. Smyrnia, |
| do. | 185 lbs. | Rhubarb, | do. London. |
| December, | 156 lbs. | Opium, | do: do. |
| do. | 1,065 lbs. | Myrrh, | do. do. |
| do. | 12,800 lbs. | Spurious Yellow Bark, | do. Santa Martha. |
| do. | 392 lbs. | Jalap, | do. Vera Cruz. |
| January, 1849, | 1,300 lbs. | Pectoral Paste, | do: San Juan. |
| do. | 2,071 lbs. | Rhubarb, | do. London. |
| do. | 3,550 lbs. | Jalap, | do. Havana. |
| do. | 1,930 lbs. | Spurious Bark, | do. Antwerp. |
| February, | 974 lbs. | Rhubarb, | do. London. |
| do. | 1,992 oz. | Iodine, | do. do. |
| March, | 1,104 oz. | Croton Oil, | do. do. |
| do. | 4,894 lbs. | Senna, | do. do. |
| do. | 1,345 lbs. | Spurious Bark, | do. do. |
| do. | 404 lbs. | Opium, | do. do. |
| do. | 1,150 lbs. | Valerian root, | do. Paris. |
| April, | 425 lbs. | Opium, | do. London. |
| do. | 1,273 lbs. | Myrrh, | do. do. |
| do. | 550 lbs. | Jalap, | do. Vera Cruz, |
| do. | 816 lbs. | do. | do. Tampico. |
| do. | 1,450 lbs. | Sarsaparilla, | do. do. |
| do. | 600 lbs. | Spurious Bark, | do. Barranquilla. |

Having in a great degree got rid of adulterated drugs from abroad, the question arises, how are we to be protected from the operations of fraudulent dealers at home? To remedy this evil, Dr. Bailey suggests that examiners should be appointed to analyse drugs at the various establishments, and to expose through the Medical Journals those which send out spurious articles. This, we think, would but partially remedy the evil. Men who are so unprincipled as to trifle with the health of the people, are sufficiently callous to care nothing for exposure, unless it touches their pockets. As a large proportion of our physicians, to their shame be it spoken—and a majority of the retailers of drugs, never read a Medical Journal, the desired information would not be sufficiently disseminated to reach the evil. We would therefore suggest that each State should pass a law visiting this offence with a heavy pecuniary punishment. In this way only can these knaves be taught that honesty is the best policy. M.

